# A self-assembled cyclodextrin nanocarrier for photoreactive squaraine

**DOI:** 10.3762/bjoc.12.248

**Published:** 2016-11-25

**Authors:** Ulrike Kauscher, Bart Jan Ravoo

**Affiliations:** 1 Organic Chemistry Institute and Center for Soft Nanoscience, Westfälische Wilhelms-Universität Münster, Corrensstrasse 40, 48149 Münster, Germany

**Keywords:** cyclodextrin, host–guest chemistry, photodynamic therapy, self-assembly, squaraine

## Abstract

Photoreactive squaraines produce cytotoxic oxygen species under irradiation and have significant potential for photodynamic therapy. Herein we report that squaraines can be immobilized on a self-assembled nanocarrier composed of amphiphilic cyclodextrins to enhance their photochemical activity. To this end, a squaraine was equipped with two adamantane moieties that act as anchors for the cyclodextrin vesicle surface. The supramolecular immobilization was monitored by using fluorescence spectroscopy and microscopy and the photochemistry of the squaraine was investigated by using absorption spectroscopy.

## Introduction

Photodynamic therapy (PDT) has become a very attractive alternative to traditional cancer therapies due to its efficiency and selectivity [1–4]. PDT is based on a photosensitizer (PS), which is delivered to cancerous tissue followed by the irradiation with light of an appropriate wavelength. Irradiation of the PS causes its transition to the excited singlet state and, via intersystem crossing; the PS reaches its triplet state. Electron transfer occurs upon relaxation to the ground state. This process can take place in two ways distinguished as type I and II mechanisms [5]. The type I mechanism describes a direct interaction between the PS and the surrounding cell material. Electrons are transferred onto the cell material, causing the production of radicals that then react with oxygen to form reactive oxygen species. The type II mechanism, however, describes the interference of the PS with the triplet ground state of oxygen, leading to the production of singlet oxygen. In both mechanisms, the PS harvests the energy of incident light to form reactive oxygen species or singlet oxygen, which are highly cytotoxic [6–7].

Most clinically used PSs are based on porphyrins, which have the disadvantage of an absorption wavelength in the range of visible light [8–9]. Visible light is scattered and absorbed by the inhomogeneous biological tissue. In order to optimise efficiency, it is vital to skew the absorption range of the PS towards the near infrared range. Then, the scattering and absorption by tissue is minimized and a more in-depth therapy becomes possible.

The search for more effective compounds with absorption in the near infrared includes amongst others cyanine [10], bodipy [11], and phthalocyanine [12] photosensitizers and has lately led to the consideration of a class of compounds known as squaraines [13–18]. Squaraines are a promising class of fluorescent dyes for PDT. In the past, squaraines showed a very low intersystem-crossing efficiency, leading to the premature conclusion that these dyes cannot be used in PDT [19]. Santos et al. showed that the intersystem-crossing efficiency of squaraines can be remarkably increased by incorporation of heavy atoms like selenium or sulfur and the addition of long aliphatic side chains [20–21]. The enhanced intersystem-crossing efficacy due to spin-orbital interactions makes modified squaraines a promising component of PDT [22–23].

Whether squaraines follow the type I or type II mechanism is discussed controversially in the literature. Santos et al. [19–20], Salice et al. [24] and Rapozzi et al. [25] investigated how benzothiazole-squaraines, which are also pursued in this project, cause cytotoxicity. Santos et al. postulated the production of singlet oxygen as a result of interaction between the squaraines and the ground state of oxygen [19–20]. A few years later, Salice et al. showed that squaraines are not efficient singlet oxygen producing agents, but can be applied successfully as singlet oxygen quenchers [23]. The authors discussed that this behaviour is based on charge-transfer processes between stacked squaraines as well as oxygen squaraine complexes. Within the same year, Rapozzi et al. described the photooxidation process of benzothiazol squaraines [24]. They showed that the irradiation of benzothiazole-squaraines results in a photodegradation process. Rapozzi et al. assume that oxidation probably involves through formation of a π-complex between the electron-rich enamine double bond and molecular oxygen. In comparison to the photo-oxidation of enaminon or other electron-rich double bonds, the complex can react further to produce 3-HBT (3-hexylbenzo[*d*]thiazol-2(3*H*)-one). The photogenerated oxidation products can then induce radical-chain reactions with the surrounding cell material, leading to cell toxicity.

**Figure 1:**
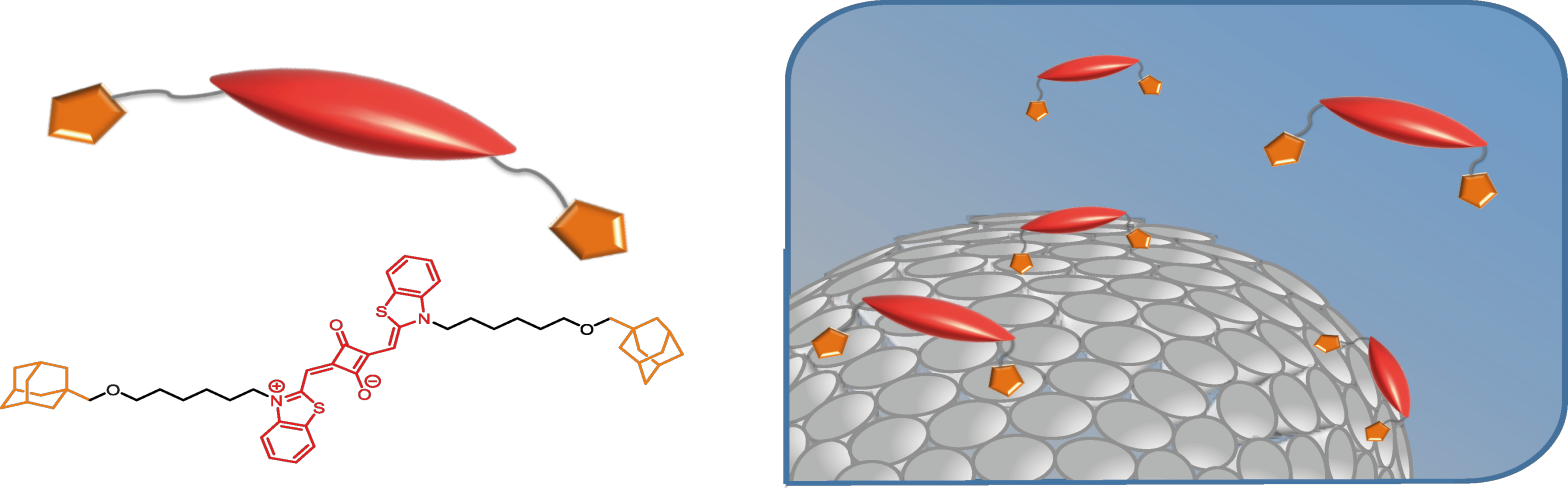
Schematic representation of adamantane-substituted squaraine (AdSq) binding as a divalent guest to the surface of cyclodextrin vesicles (CDV).

However, like most PS, squaraines have poor water solubility, which leads to aggregation and inactivation under physiological conditions, causing a reduced production of reactive oxygen species [26]. To overcome this drawback, we considered to immobilize the squaraines onto a platform that provides spatial separation (Figure 1). Our platforms of choice are vesicles self-assembled from amphiphilic cyclodextrin [27]. Given their negligible toxicity cyclodextrins have been utilized as carriers in a number of studies [28–29]. Amphiphilic cyclodextrins substituted with hydrophobic alkyl groups on the primary side and hydrophilic oligo(ethylene glycol) units on the secondary side can be synthesized in a simple and straightforward three step synthesis [27,30]. Cyclodextrin vesicles (CDVs) can be easily prepared by sonication or extrusion in aqueous solution. CDVs have the unique possibility of versatile surface decoration by the simple addition of guest molecules to the aqueous solution of the vesicle [30]. The hydrophobic cyclodextrin cavities are well known to form size-selective inclusion complexes with apolar compounds such as adamantane [31]. In several publications, we have shown that CDVs retain this ability by keeping the cavity available for host–guest complexation. In that way, it was possible to decorate CDVs with carbohydrates [32–33], with peptides [34], and also with DNA [35]. Moreover CDVs were also shown to form very dense membranes in combination with phospholipids and cholesterol [36]. Previous studies also showed promising results for PDT applications when a well-known PS, phthalocyanine, was used to decorate the cyclodextrin vesicles [37–38]. The immobilization led to an increased photoactivity of the phthalocyanines due to suppression of aggregation and inactivation of the PS at the surface of the CDVs.

In this contribution we show that squaraines can also be immobilized onto CDVs. For this purpose, the squaraines are equipped with two adamantane functions that bind into the cavities of cyclodextrin on the surface of the CDVs (Figure 1). The resulting divalent host–guest interaction should lead to a higher binding affinity of the squaraine for the CDV without affecting its photochemistry. In addition, it is expected that the aggregation of squaraine will be supressed by immobilization at the CDV surface. Ultimately, the photoactive squaraine can be combined with other functional guests, such as targeting units and tracers, to further enhance the potential of the nanocarrier.

## Results and Discussion

Adamantane-substituted squaraine (AdSq) was synthesized in a three step synthesis. Benzothiazole rings were introduced to enlarge the π-system. The sulfur atoms of these moieties increase the intersystem crossing due to the heavy atom effect. Additionally hexyl alkane chains were introduced on both N-termini. Santos et al. showed that these lead to a higher intersystem crossing compared to shorter alkane chains [20–21]. The analytical data for AdSq (see Supporting Information File 1) are consistent with the molecular structure shown in Figure 1.

**Figure 2:**
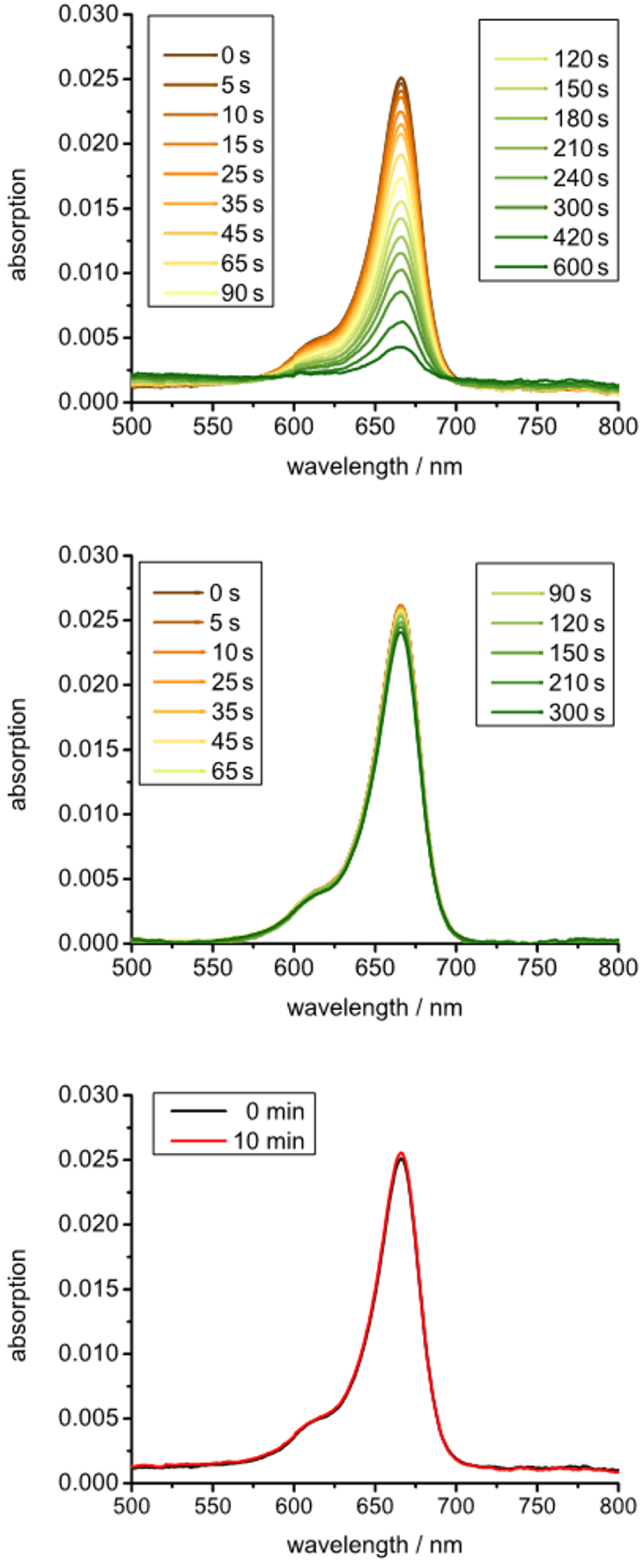
Absorption spectra of AdSq in acetonitrile. [AdSq] = 7.5 µM. Top: Absorption at different time points during irradiation. Middle: Absorption at different time points during irradiation of an argon equilibrated solution. Bottom: Absorption after and before storing in the dark for 10 min.

In a first set of experiments, we investigated the photochemical properties of AdSq in acetonitrile (Figure 2). The absorption spectrum shows a peak at 665 nm indicative of the presence of squaraine monomers in the solution. A shoulder around 610 nm indicates aggregation of the squaraine due to π–π stacking. A comparison of absorption spectra in different solvents revealed that the amount of aggregation is rather low in acetonitrile or ethanol but increases dramatically if water is used as a solvent (see Figure S1 in Supporting Information File 1). This is in agreement with literature and highlights the importance of introducing a platform for the immobilization of the squaraines as to provide steric separation and suppress aggregation.

In the next step, we investigated the photoactivation under irradiation. To this end, a solution of AdSq was irradiated with light of a wavelength higher than 630 nm (250 W halogen lamp, long pass filter 630 nm). Absorption spectra were taken at different time points of irradiation. The obtained data show a decay of absorption with time of irradiation (Figure 2, top). Clearly, AdSq is undergoing a reaction under irradiation which leads to its decomposition. To verify that the proposed mechanism by Rapozzi (see above) is correct we carried out further experiments. If the solution of AdSq in acetonitrile was equilibrated in an argon atmosphere to drive away any oxygen, the absorption spectra obtained show the same absorption intensity for all time points (Figure 2, middle). This reveals that AdSq undergoes no decomposition in absence of oxygen and that photodecomposition is definitely due to interaction with oxygen. To test if the decomposition mechanism is light driven, another set of absorption spectra were taken in an oxygen-equilibrated solution that was stored in the dark for 10 min. In this case, the absorption spectra show no decrease indicating that again no decomposition is obtained (Figure 2, bottom). Thus, AdSq is stable in solution if stored in the dark.

Next, we investigated the immobilization of AdSq by host–guest interaction with CDVs. Amphiphilic β-cyclodextrins substituted with 7 dodecylsulfide groups on the primary side and 7 oligo(ethylene glycol) units on the secondary side were obtained via a straightforward three step synthesis as described [27,30]. A thin film of these amphiphiles was obtained by evaporation of a chloroform solution in a round bottom flask. Hydration and extrusion with a Liposofast extruder and membranes with 100 nm pore size yield bilayer vesicles of an approximate size of 100 nm. CDVs were decorated with AdSq by simple addition of the AdSq to the aqueous dispersion of the CDVs.

**Figure 3:**
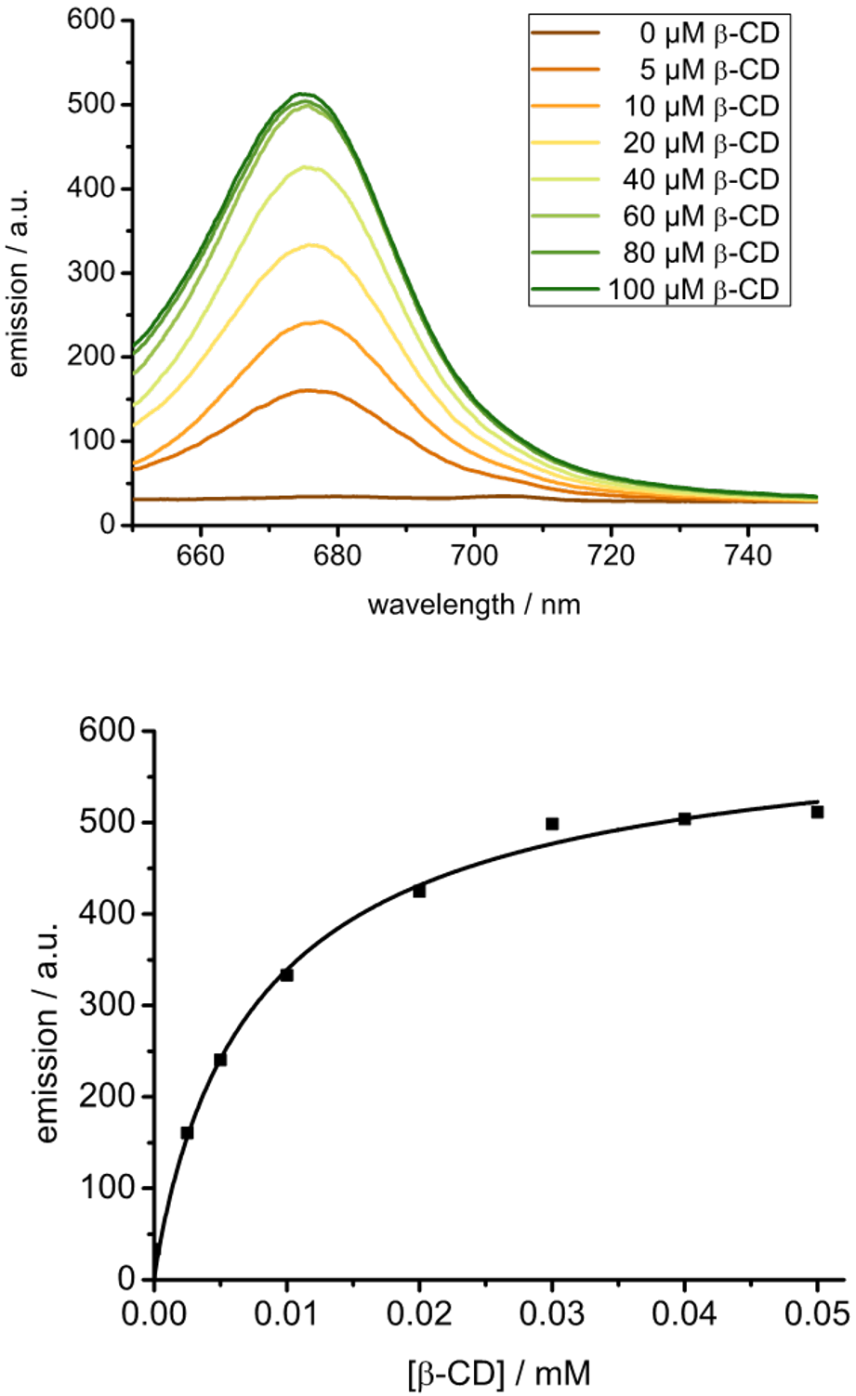
Top: Emission spectra (ex: 630 nm) of AdSq immobilized at CDV. [CDV] = 0–100 µM; [AdSq] = 5 µM. Bottom: Langmuir regression of a fluorescence titration of CDV to AdSq. [AdSq] = 10 µM, [CDV] = 0–100 µM. It is assumed that only cyclodextrins on the outer surface of the CDV are available for host–guest interactions.

Interestingly, the immobilization of AdSq on CDV can be directly observed by fluorescence spectrometry. To this end, solutions with different concentrations of CDV were prepared. AdSq was dissolved in acetonitrile in the dark and added to the vesicle solution. The percentage of acetonitrile in water was kept under 0.5%, so that any influence on solubility of AdSq, stability of CDVs or host guest interaction can be neglected. Fluorescence spectra were taken 20 min after addition of AdSq to assure the complete equilibration of the CDV dispersion. The fluorescence spectrum of AdSq without CDV shows no fluorescence, while increasing fluorescence intensity was detected with more CDV available (Figure 3, top). These results indicate that immobilization of the AdSq on the surface of CDV suppresses the aggregation of AdSq in aqueous solution and that the AdSq fluorescence is enhanced due to a suppression of intermolecular π–π stacking. The more CDV is available, the more AdSq is immobilized on the surface of the vesicles. To support this explanation, absorption spectra were taken of the solutions that were prepared for the fluorescence experiments. The absorption spectra show a decrease of the signal at 615 nm that indicates the presence of the aggregated form of AdSq (see Figure S2 in Supporting Information File 1).

The fluorescence spectra show a saturation of the intensity maximum for a concentration of 60 µM CDV. Assuming that the accessible concentration of cyclodextrin available for the complexation of guest is only 30 µM (since approximately 50% of the amphiphilic cyclodextrins reside at the interior surface of the CDV and AdSq is too large and too polar to pass the membrane), saturation is reached at a six-fold excess of host to guest, even though both are present in micromolar concentrations only. The binding constant of AdSq with the cyclodextrins in the CDV could be obtained by plotting the maximum fluorescence emission (at 676 nm) against the concentration of available cyclodextrin (Figure 3, bottom). The resulting Langmuir isotherm was fitted by linear regression (see Supporting Information File 1 for details) and the binding constant was determined to be *K*_a_ = 1.2 × 10^5^ M^−1^. The high-affinity binding of AdSq is a clear indication that both adamantanes are involved in binding to the CDV and that the interaction is effectively divalent.

**Figure 4:**
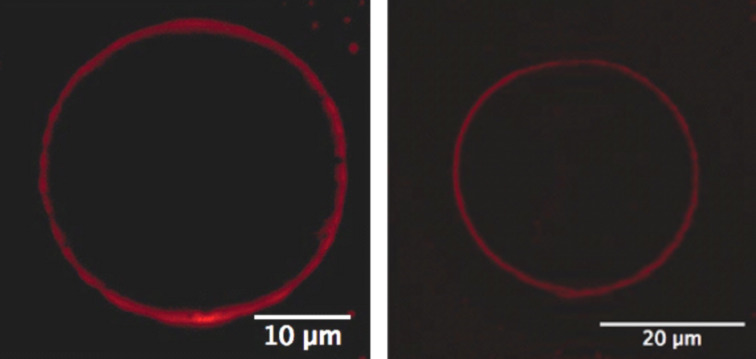
Confocal fluorescence microscopy of giant unilamellar vesicles (GUVs) of amphiphilic cyclodextrins with AdSq on their outer surface.

In another set of experiments, giant unilamellar vesicles (GUVs) of amphiphilic cyclodextrins were formed by electroformation [35]. The obtained vesicles have a size of several micrometers and are thus directly visible by fluorescence microscopy. AdSq were added to a solution of the GUVs and immobilization of the molecules was examined with confocal fluorescence spectroscopy. The images taken show GUVs with a red luminescent membrane due to the squaraines bound to the GUV surface (Figure 4). Thus, fluorescence microscopy provided direct evidence for the immobilization of AdSq on the CDV.

**Figure 5:**
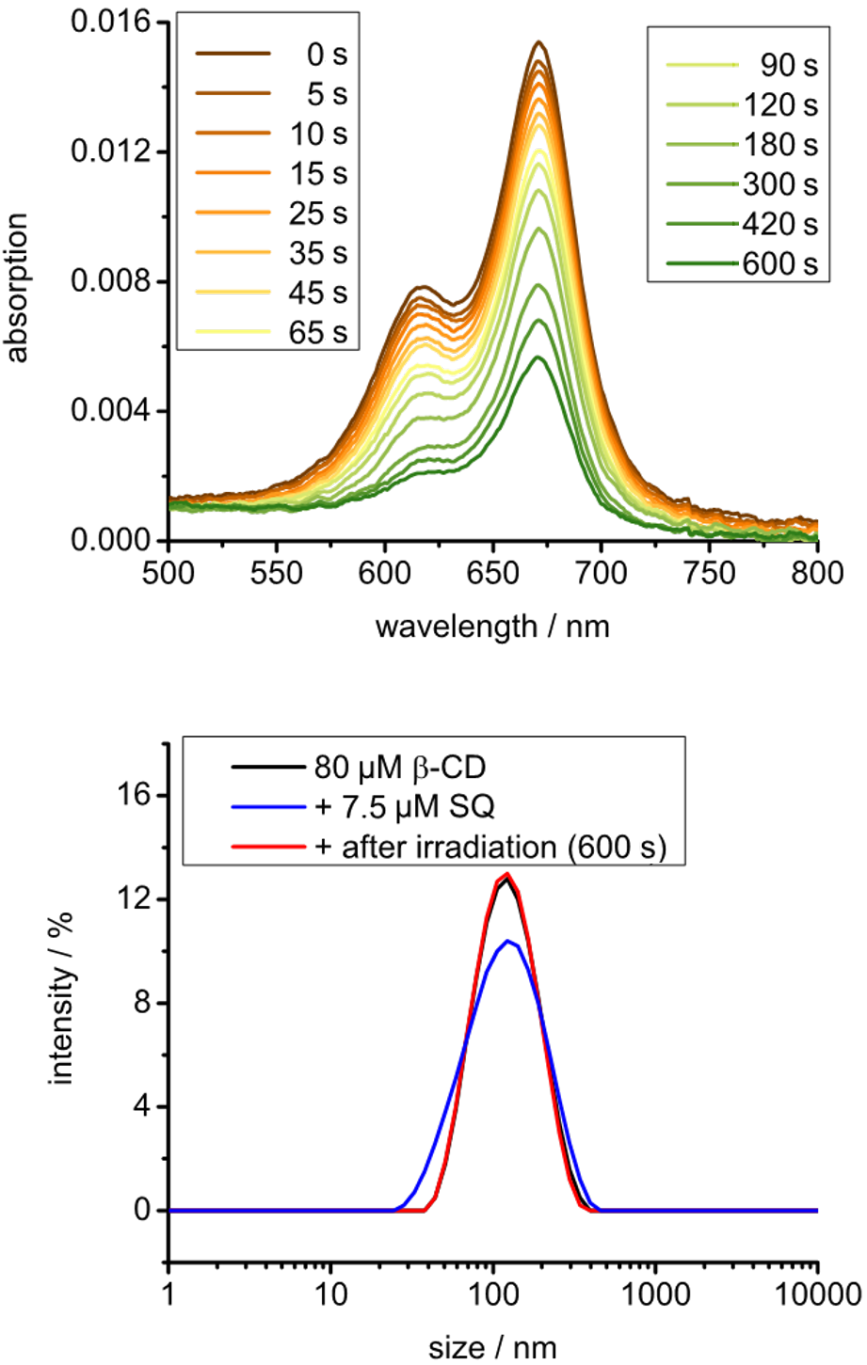
Top: Absorption spectra at different time points during irradiation of a CDV solution with AdSq immobilised on the vesicle surface. Bottom: DLS spectra before and after irradiation. [AdSq] = 7.5 µM, [CDV] = 80 µM.

After the immobilization of AdSq on the surface of CDV was demonstrated, we investigated the photochemistry of AdSq on CDV. To this end, AdSq was added to an aqueous dispersion of CDV exactly as described above. The solution was irradiated for 600 s and absorption spectra were taken at various time points. As shown before in the acetonitrile solution, also AdSq immobilized on CDV photodecomposition (Figure 5). The decomposition of AdSq on the surface of CDV versus its decomposition in acetonitrile solution was compared (see Figure S3 in Supporting Information File 1). The immobilized squaraines showed a slower decay indicating a higher stability of squaraines on the CDV. To verify that the CDV are stable during this process, DLS spectra were taken. These reveal the same size for the vesicles before and after addition of squaraines as well as after irradiation, so that significant photodamage of the nanocarrier can be excluded. We note that our measurements are performed under atmospheric conditions. At these conditions the concentration of oxygen in water is 1.25 mM while the concentration of oxygen in acetonitrile is between 8.1–12.1 mM, depending on the type of measurement. [39]. The lower oxygen concentration in water can enhance the lifetime of AdSq on CDV.

## Conclusion

We demonstrated the successful synthesis of a symmetric benzothiazol squaraine (AdSq) substituted with two adamantane groups. We showed that this squaraine can be activated by irradiation and undergoes an oxygen-dependent decomposition reaction as proposed by Rapozzi et al. for similar squaraines [25]. Host–guest inclusion between the adamantane groups and the cyclodextrin cavities at the surface of cyclodextrin vesicles (CDVs) results in efficient immobilization of the squaraine on a nanocarrier. In addition, π–π stacking (and hence inactivation) of the squaraine is supressed due to the steric separation of the squaraines on the vesicle surface. In short, the self-assembled nanosystem has several advantageous properties to be used as a PDT system. We envision that the photoactive squaraine can be combined with other functional guests, such as targeting units and tracers, to enhance the potential of the nanocarrier. Further studies have to be carried out to reveal the effectivity in medical applications.

## Experimental

**Figure 6:**
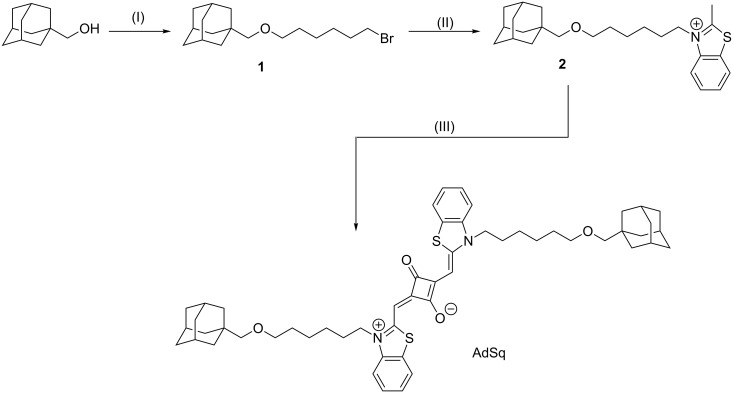
Synthesis of AdSq (I) NaH, DMF, 1,6-dibromohexane, 24 h, rt. (II) benzothiazole, acetonitrile, 12 h, 90 °C, 5%; (III) squaric acid, benzene, *n*-butanol, pyridine, 12 h, 120 °C, 26%.

In the following the synthesis of AdSq is described (Figure 6). NMR spectra can be found in Supporting Information File 1. In the cases where the experimental protocol required an inert gas atmosphere, the Schlenk method was used under argon atmosphere.

**1-[{(6-Bromohexyl)oxy}methyl]adamantane (1)**


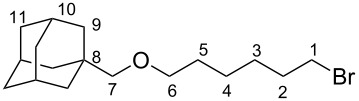


Adamantanyl-1-methanol (4.98 g, 29.95 mmol) and NaH (wt 60%, 2.38 g) were dissolved in dry DMF and stirred at room temperature for 90 minutes. 1,6-Dibromohexane (18.27 mL, 120 mmol) was then added drop-wise to the reaction mixture. The reaction mixture was stirred at room temperature for 24 hours and the excess NaH was subsequently quenched by addition of water. The product was then extracted from pentane (1×) and water (3×). The product was dried over MgSO_4_ and excess solvent was removed by rotary evaporation. A fraction of crude product was then purifiied through silica column chromatography (EtOAc/cyclohexane (40:1), *R*_f_: 0.6). Molecular formula: C_17_H_29_BrO. ^1^H NMR (400 MHz, CDCl_3,_ 298 K) δ 3.33 (m, 4H, 1,6-H), 2.88 (s, 2H, 7-H), 1.89 (p, *J* = 3.1 Hz, 3H, 10-H), 1.84–1.75 (m, 2H, 2-H ), 1.68–1.55 (m, 6H, 11-H), 1.54-1.25 (m, 14H, 2-5-9-H) ppm; ^13^C NMR (75 MHz, CDCl_3,_ 298 K) δ 81.92 (CH_2_, 7-C), 71.40 (CH_2_, 6-C), 39.74 (3 CH_2_, 9-C), 37.24 (3 CH_2_, 11-C), 34.08 (C_q_, 8-C), 33.96 (CH_2_, 1-C), 32.78 (CH_2_, 2-C), 29.38 (CH_2_, 5-C), 28.29 (3 CH, 10-C), 28.01 (CH_2_, 3-C), 25.37 (CH_2_, 4-C) ppm; HRMS–ESI (*m*/*z*): calcd for [C_17_H_29_BrO]^+^: 351.1294; found: 351.1299.

**3-[6-{(Adamantan-1-yl)methoxy}hexyl]-2-methylbenzo[*d*]thiazole (2)**


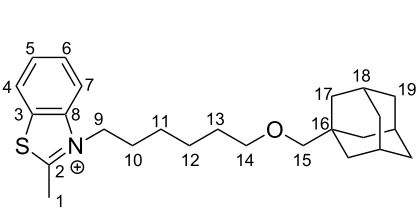


Methylbenzothiazole (0.89 g, 6 mmol) and 1-[{(6-bromohexyl)oxy}methyl]adamantane (**1**, 1.97 g, 6 mmol) were dissolved in 4 mL of acetonitrile and the resulted solution was stirred at 90 °C overnight. Afterwards the solvent was evaporated by rotary evaporation. The residue was washed with diethyl ether. The product was filtered and the precipitate was washed with diethyl ether. Afterwards the precipitate was redissolved in ethanol. The solvent was evaporated and the product was obtained as a dark red solid (0.119 g , 0.3 mmol, 5%). Molecular formula: C_25_H_36_NOS; ^1^H NMR (300 MHz, CDCl_3,_ 298 K) δ 8.38 (m, 1H, 7-H), 7.98 (d, *J* = 8.5 Hz, 1H, 4-H), 7.75 (dd, *J* = 8.5, 1.3 Hz, 1H, 6-H), 7.71–7.56 (m, 1H, 5-H), 4.88 (t, *J* = 7.8 Hz, 2H, 9-H), 3.44 (s, 3H, 1-H), 3.32 (t, *J* = 6.0 Hz, 2H, 14-H), 2.90 (s, 2H, 15-H), 1.91 (m, 10-/18-H), 1.68–1.44 (m, 16H, 11-/12-/13-/17-/19-H), 1.22 (m, 2H) ppm; ^13^C NMR (75 MHz, CDCl_3,_ 298 K) δ 175.66 (C_q,_ 2-C), 140.97 (C_q_, 8-C), 129.88 (C_q_, 3-C), 129.42, 128.58, 125.03, 116.40 (CH, 4-7-C), 82.02 (CH_2_, 15-C), 71.24 (CH_2_, 14-C), 51.04 (CH_2_, 9-C), 39.81 (3 CH_2_, 17-C), 37.29 (3 CH_2_, 19-C), 34.16 (C_q_, 16-C), 29.38 (CH_2_, 13-C), 28.81 (CH_2_, 10-C), 28.33 (3 CH, 18-C), 26.69 (CH_2_, 11-C), 25.93 (CH_2_, 12-C), 19.06 (CH_3_, 1-C) ppm; HRMS–ESI (*m*/*z*): calcd for [C_25_H_36_NOS]^+^: 398.2512; found: 398.2516.

**2-((3-(6-((Adamantan-1-yl)methoxy)hexyl)benzo[*d*]thiazol-2(3*H*)-ylidene)methyl)-4-((3-(6-((adamantan-1-yl)methoxy)hexyl)benzo[*d*]thiazol-3-ium-2-yl)methylene)-3-oxocyclobut-1-en-1-olate, squaraine (AdSq)**


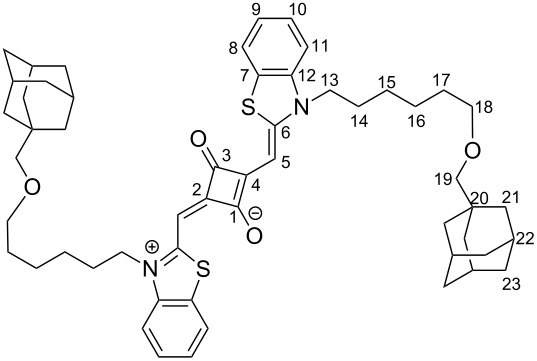


All solvents were dried and equilibrated with argon. The reaction was performed in the dark. Squaric acid (10 mg, 0.09 mmol) and compound (**2**, 70 mg, 0.18 mmol) were dissolved in a mixture of benzene, *n*-butanol and pyridine (2:1:1). The reaction was stirred for 12 h at 120 °C. The crude product was concentrated in vacuo and purified by column chromatography under argon atmosphere (DCM:MeOH 9:1, *R*_f_: 0.6). The product was obtained as a dark blue solid (20 mg, 0.088 mmol, 26%). Molecular formula: C_54_H_68_N_2_O_4_S_2_. ^1^H NMR (600 MHz, CDCl_3,_ 298 K) δ 9.41 (dd, *J* = 18.1, 6.1 Hz, 2H, H_ar_), 8.97–8.91 (m, 1H, H_ar_), 8.55–8.45 (m, 2H, H_ar_), 8.13 (t, *J* = 6.9 Hz, 2H, H_ar_), 8.02 (dd, *J* = 8.0, 5.5 Hz, 1H, H_ar_), 6.34 (s, 1H, 5-H), 4.96 (dd, *J* = 8.8, 6.2 Hz, 2H, 13-H), 4.63 (td, *J* = 6.6, 1.3 Hz, 1H, 5-H), 4.19 (qd, *J* = 11.0, 6.0 Hz, 2H, 13-H), 3.39–3.26 (m, 4H, 18-H), 2.94–2.89 (m, 4H, 19-H), 2.14–1.03 (m, 42H, 14-,16-,17-,21-,22-,23-H), 1.02–0.81 (m, 4H, 15-H) ppm; ^13^C NMR (151 MHz, CDCl_3,_ 298 K) δ 198.35 (Cq, 3-C), 190.29 (Cq, 1-C), 187.25 (Cq, 6-C), 167.87 (Cq, 6-C), 145.25 (C_q_, 2-C), 141.94, 140.97 (2CH, 12-C), 132.55, 130.99 (C_q_, 7-C), 128.89, 128.59, 127.72, 127.27 (4CH, 9-,10-C), 124.95, 122.46 (CH, 8-,11-C), 112.36 (2CH, 5-C), 82.02 (2CH_2_, 19-H), 73.01, 71.44, 71.31 (2CH_2_, 18-C), 62.34 (CH, 13-C), 47.01 (CH, 13-C), 39.86 (CH, 21-C), 37.34 (CH, 23-C), 34.20 (C_q_, C-20), 32.05, 31.99 (2CH_2_, 17-C), 29.47, 29.38 (2CH_2_, 14-C), 28.38 (2CH, 22-C), 26.09, 26.02 (2CH_2_, 15-C), 25.99, 25.76 (2CH_2_, 16-C), 18.69 ppm; MALDI–MS (*m*/*z*): calcd for [C_54_H_68_N_2_O_4_S_2_]^+^: 872,46; found: 872,47.

## Supporting Information

File 1 Additional measurements and methods.

## References

[1] Bonnett R (2000). Chemical Aspects of Photodynamic Therapy.

[2] Bonnett R (1995). Chem Soc Rev.

[3] Robertson C A, Evans D H, Abrahamse H (2009). J Photochem Photobiol, B.

[4] Maisch T (2009). Mini-Rev Med Chem.

[5] Felsher D W (2003). Nat Rev Cancer.

[6] Josefsen L B, Boyle R W (2008). Met-Based Drugs.

[7] Ortel B, Shea C R, Calzavara-Pinton P (2009). Front Biosci.

[8] Detty M R, Gibson S L, Wagner S J (2004). J Med Chem.

[9] Cherno N K, Krusir G V (2005). Prikl Biokhim Mikrobiol.

[10] Delaey E, van Laar F, De Vos D, Kamuhabwa A, Jacobs P, de Witte P (2000). J Photochem Photobiol, B.

[11] Awuah S G, Polreis J, Biradar V, You Y (2011). Org Lett.

[12] Jiang Z, Shao J, Yang T, Wang J, Jia L (2014). J Pharm Biomed Anal.

[13] Sreejith S, Divya K P, Ajayaghosh A (2008). Angew Chem, Int Ed.

[14] Law K Y (1987). J Phys Chem.

[15] Emmelius M, Pawloski G, Vollmann H W (1989). Angew Chem, Int Ed.

[16] Law K Y (1993). Chem Rev.

[17] Dolmans D E J G J, Fukumura D, Jain R K (2003). Nat Rev Cancer.

[18] Yagi S, Nakazumi H, Kim S-H (2006). Syntheses and Applications of Squarylium Dyes. Functional Dyes.

[19] Kamat P V, Das S, Thomas K G, George M V (1992). J Phys Chem.

[20] Santos P F, Reis L V, Almeida P, Oliveira A S, Vieira Ferreira L F (2003). J Photochem Photobiol, A.

[21] Santos P F, Reis L V, Almeida P, Serrano J P, Oliveira A S, Vieira Ferreira L F (2004). J Photochem Photobiol, A.

[22] Foote C S, Lin J W-P (1968). Tetrahedron Lett.

[23] Mazur S, Foote C S (1970). J Am Chem Soc.

[24] Salice P, Arnbjerg J, Pedersen B W, Toftegaard R, Beverina L, Pagani G A, Ogilby P R (2010). J Phys Chem A.

[25] Rapozzi V, Beverina L, Salice P, Pagani G A, Camerin M, Xodo L E (2010). J Med Chem.

[26] Monge-Fuentes V, Muehlmann L A, de Azevedo R B (2014). Nano Rev.

[27] Ravoo B J, Darcy R (2000). Angew Chem, Int Ed.

[28] Chauhan P, Hadad C, Sartorelli A, Zarattini M, Herreros-López A, Mba M, Maggini M, Prato M, Carofiglio T (2013). Chem Commun.

[29] Uekama K, Hirayama F, Irie T (1998). Chem Rev.

[30] Falvey P, Lim C W, Darcy R, Revermann T, Karst U, Giesbers M, Marcelis A T M, Lazar A, Coleman A W, Reinhoudt D N (2005). Chem – Eur J.

[31] Chen Y, Liu Y (2010). Chem Soc Rev.

[32] Voskuhl J, Stuart M C A, Ravoo B J (2010). Chem – Eur J.

[33] Kauscher U, Ravoo B J (2012). Beilstein J Org Chem.

[34] Versluis F, Tomatsu I, Kehr S, Fregonese C, Tepper A W J W, Stuart M C A, Ravoo B J, Koning R I, Kros A (2009). J Am Chem Soc.

[35] Nalluri S K M, Voskuhl J, Bultema J B, Boekema E J, Ravoo B J (2011). Angew Chem, Int Ed.

[36] Kauscher U, Stuart M C A, Drücker P, Galla H-J, Ravoo B J (2013). Langmuir.

[37] Voskuhl J, Kauscher U, Gruener M, Frisch H, Wibbeling B, Strassert C A, Ravoo B J (2013). Soft Matter.

[38] Galstyan A, Kauscher U, Block D, Ravoo B J, Strassert C A (2016). ACS Appl Mater Interfaces.

[39] Li Q, Batchelor-McAuley C, Lawrence N S, Hartshorne R S, Compton R G (2013). J Electroanal Chem.

