# Supplementary material for: A self-assembled cyclodextrin nanocarrier for photoreactive squaraine
Source: Beilstein J Org Chem. 2016 Nov 25;12:2535–42. doi: 10.3762/bjoc.12.248 (PMC5238535; doi:10.3762/bjoc.12.248)
Supplement: File 1 — Additional measurements and methods. [file Beilstein_J_Org_Chem-12-2535-s001.pdf]

Supporting Information

for

A self-assembled cyclodextrin nanocarrier for photoreactive squaraine

Ulrike Kauscher and Bart Jan Ravoo*

Address: Organic Chemistry Institute and Center for Soft Nanoscience, Westfälische Wilhelms-Universität Münster, Corrensstrasse 40, 48149 Münster (Germany)

Email: Bart Jan Ravoo - b.j.ravoo@uni-muenster.de

*Corresponding author

Table of contents:

1) **Additional measurements**
2) **Methods**

1) Additional measurements

UV–vis absorption spectra were taken of AdSq dissolved in different solvents (Figure S1). The absorption curves obtained show a strong absorption between 650–680 nm together with a smaller absorption signal between 600–620 nm. The signal between 650–680 nm is characteristic of squaraine monomers while the second, smaller, signal is caused by aggregated squaraines due to π–π stacking. The wavelength of the maxima is dependent on the polarity of the solvent used. In the case of water, the smaller absorption signal is significantly enhanced in comparison to the other solvents used.

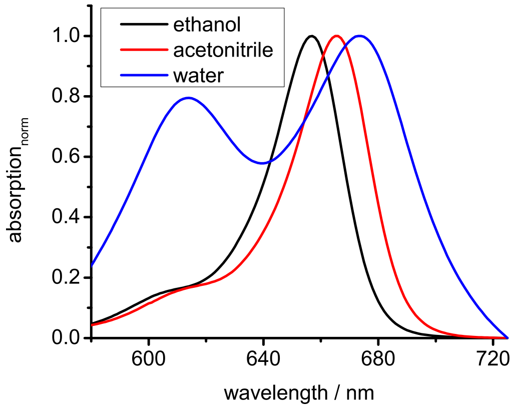

Figure S1: Normalized absorption spectrum of AdSq in ethanol, acetonitrile and water.

AdSq can be immobilized on CDV. With increasing concentration of CDV added to the squaraine solution, additional squaraine is immobilized leading to less aggregation. The signal between 600–620 nm is thus suppressed relative to the peak at at 650–680 nm (Figure S2). Note that Figure S2 shows normalized data.

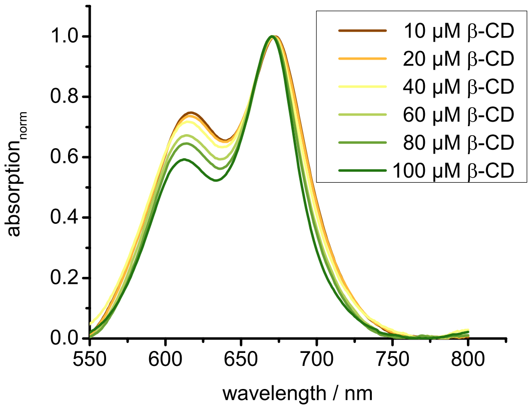

Figure S2: Normalized absorption spectra of AdSq in the presence of CDV. [CDV] = 0–100 µM; [AdSq] = 5 µM.

The decomposition of AdSq on the surface of CDV versus its decomposition in acetonitrile solution was compared. The decrease of the absorption maxima was followed over time after irradiation. Exponential curves were obtained in both cases over the course of 600 s. The immobilized squaraines have a slower decay indicating a higher stability of squaraines on the surface of the vesicles.

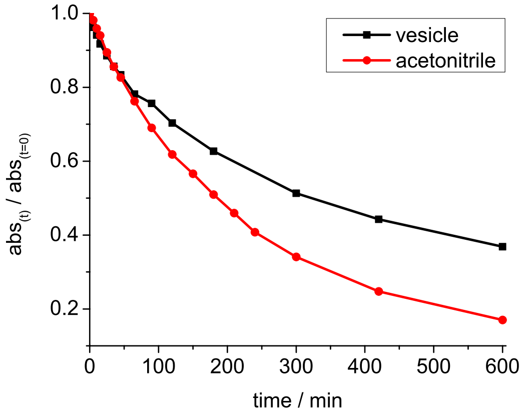

Figure S3: Absorption maximum of AdSq at 665 nm (acetonitrile) or 667 nm (solution of CDV) at different time points of irradiation.

Additional experiments were carried out to demonstrate that no singlet oxygen is produced. To this end anthracenediylmethylene malonic acid (ADMDM) was added to a solution of AdSq immobilized on CDV. The fluorescence of ADMDM is measured while the sample is irradiated (Figure S3). ADMDM is a singlet oxygen sensitive fluorescent molecule. It reacts with singlet oxygen to form the non-fluorescent endoperoxide. The fluorescence of ADMDM does not decrease over time, serving as evidence that no singlet oxygen is present in solution.

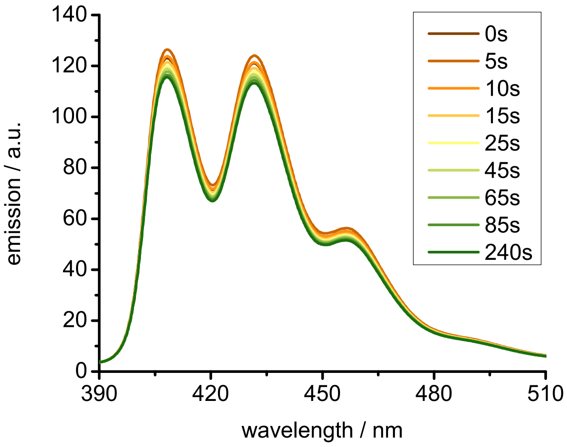

Figure S4: Emission spectra (Ex = 370 nm) of a solution of ADMDM and AdSq in a CDV solution at different time points of irradiation. [ADMDM] = 10 µM; [AdSq] = 10µM; [CDV] = 200 µM.

2) Methods:

Preparation of squaraine decorated vesicles:

Vesicles were prepared via hydration of a lipid film followed by extrusion. A 1 mM stock solution of the amphiphilic β-cyclodextrin was prepared in chloroform. The desired amount of β-cyclodextrin was then pipetted into a round bottom flask and the solvent was removed with an argon stream. The remaining lipid film was hydrated and afterwards extruded with a *Lisposofast* Extruder through a polycarbonate membrane with a pore size of 100 nm. A 1 mM solution of squaraine was prepared in acetonitrile. 5 µL of this solution were added to 995 µL of the vesicle solution.
GUVs were prepared via electroformation. 16 µL of the previously prepared stock solution of β-CD were carefully placed on an ITO coated glass slide. The solvent was evaporated in a vacuum oven at 50 °C. The lipid film was hydrated with a buffer solution (1 mM HEPES, 100 succrose, pH 7.5). An alternating electric field was applied with 1 V and 10 Hz at 50 °C for 1 h.

Irradiation experiments:

For the irradiation of samples with light of a wavelength higher 630 nm a projector with a 250 W halogen lamp (*Agfa*) was used. The light was filtered with a long pass filter (<630 nm).

UV–vis measurements:

UV–vis spectra were recorded on a double beam spectrometer (*Jasco V650, Jasco Analytical Instruments, Easton, USA)*. Samples were measured in 1.5 mL disposable semi-micro cuvettes with dimensions 12.5 × 12.5 × 45 mm and 10 mm path with Mili-Q water as an eluent or in a Semi-Micro Cell *108F-QS (Hellma Analytics, Mühlheim, Deutschland*) if organic solvents (acetonitrile) were used.

Dynamic light scattering (DLS)

DLS was recorded on *Malvern Nano Zetasizer (Malvern Instuments Ltd., Worcestershire, UK).* The sizes of particles were measured in low volume disposable PMMA cuvettes. Water was used as an eluent. *Dispersion Technology Software (Malvern Instruments Ltd., Worcestershire, UK, Version 6.20)* was used to analyze data.

Fluorescence microscopy

GUVs decorated with Squarains were investigated with *Axio Observer Research Microscope (Carl Zeiss Microscopy GmbH, Jena)* with an *High-End Fluorescence-Imaging-System ApoTome.2 (Carl Zeiss Microscopy GmbH, Jena)* on BSA coated slides.

Fluorescence measurements

Spectra were obtained through a *JASCO FP6500 Spectrofluorometer (JASCO, Easton, Netherlands)* with an excitation wavelength of 630 nm. Samples were measured in a Semi-Micro Fluorescence Cell *108F-QS (Hellma Analytics, Mühlheim, Deutschland*). 5 µL of a concentrated squaraine solution in acetonitrile (1 mM) were added to 995 µL of aqueous vesicle solution. The sample was incubated in the dark and then measured. This procedure was repeated for each vesicle concentration. The following equation was used to fit the fluorescence titration:

$$y = V\mathrm{max}*[\mathrm{CD}]^{\mathrm{n}}/(K_d^{\,n}*[\mathrm{CD}]^{\mathrm{n}}),$$

where y is the emission intensity at certain ratios of host to guest, Vmax is the maximum emission extrapolated to infinite CD concentration, [CD] is the CD concentration and n is a measure of cooperativity. Origin software was used for the fitting.

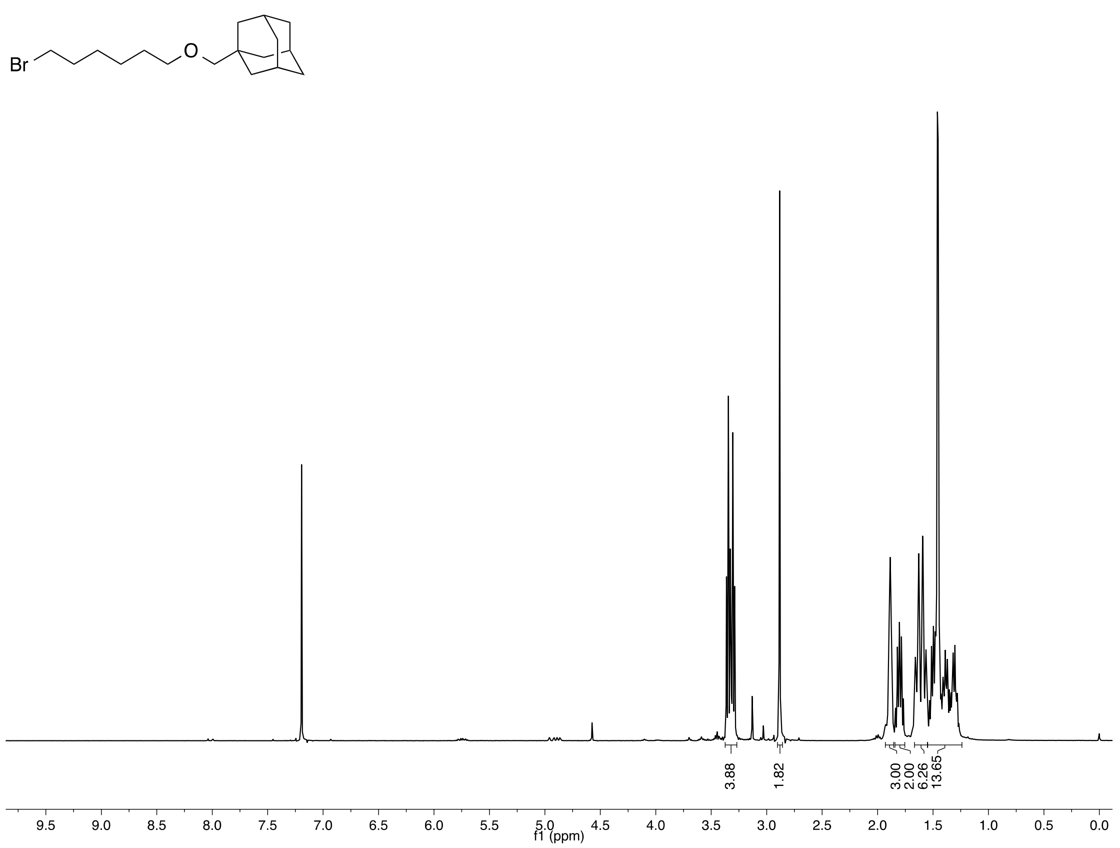

Figure S5: ^{1}H NMR of compound **1** in $CDCl_3$ at 298 K.

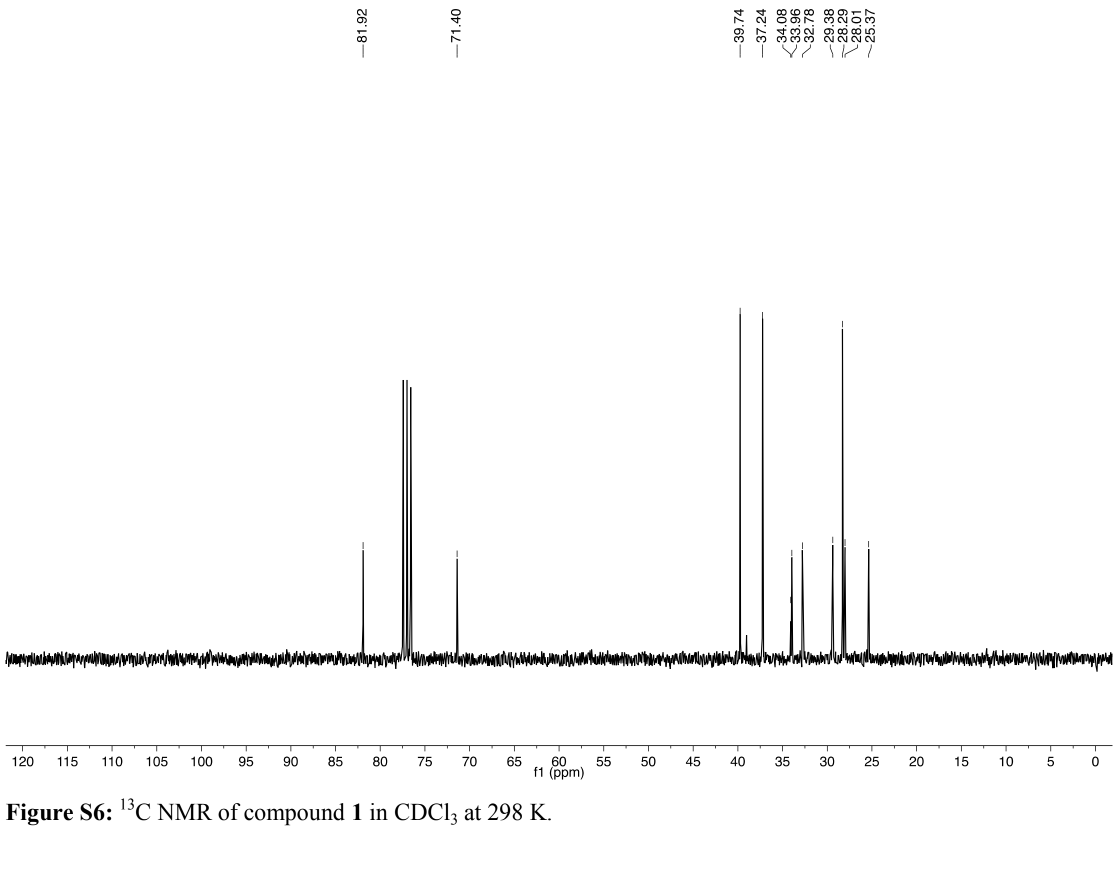

Figure S6: ^{13}C NMR of compound **1** in $CDCl_3$ at 298 K.

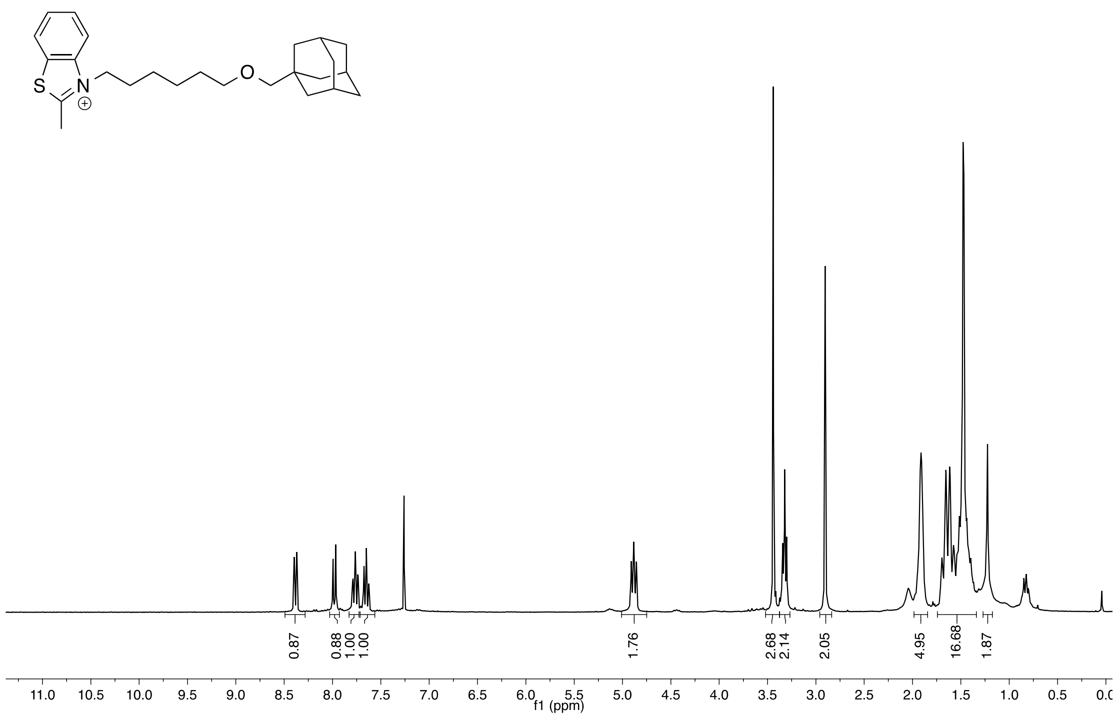

Figure S7: ^{1}H NMR of compound **2** in $CDCl_3$ at 298 K.

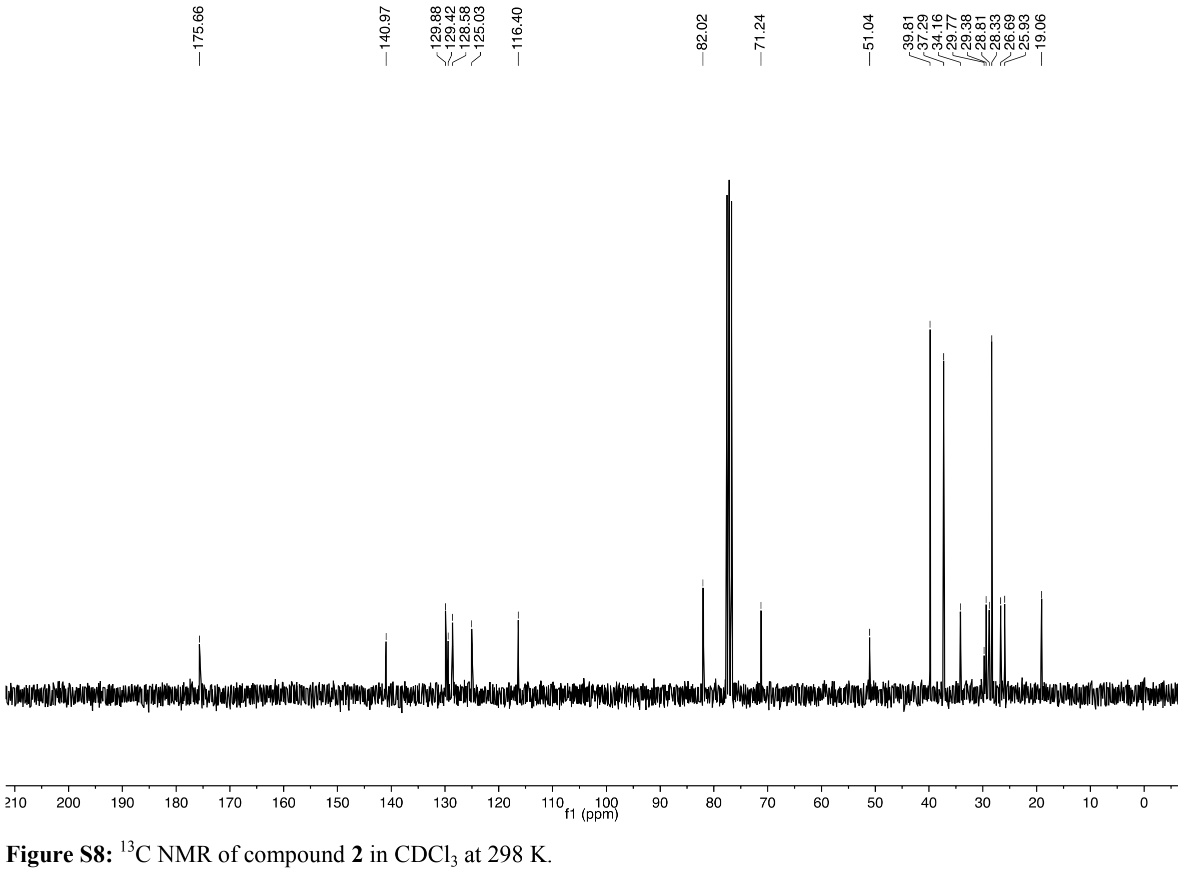

Figure S8: ^{13}C NMR of compound **2** in $CDCl_3$ at 298 K.

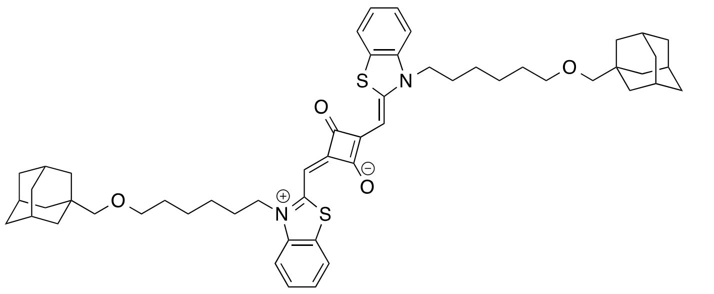

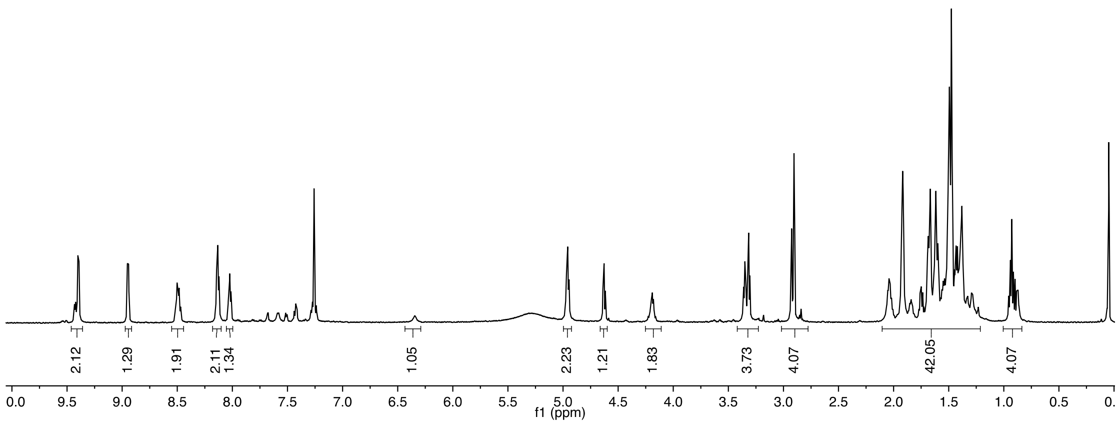

Figure S9: ^{1}H NMR of AdSq in $CDCl_3$ at 298 K.

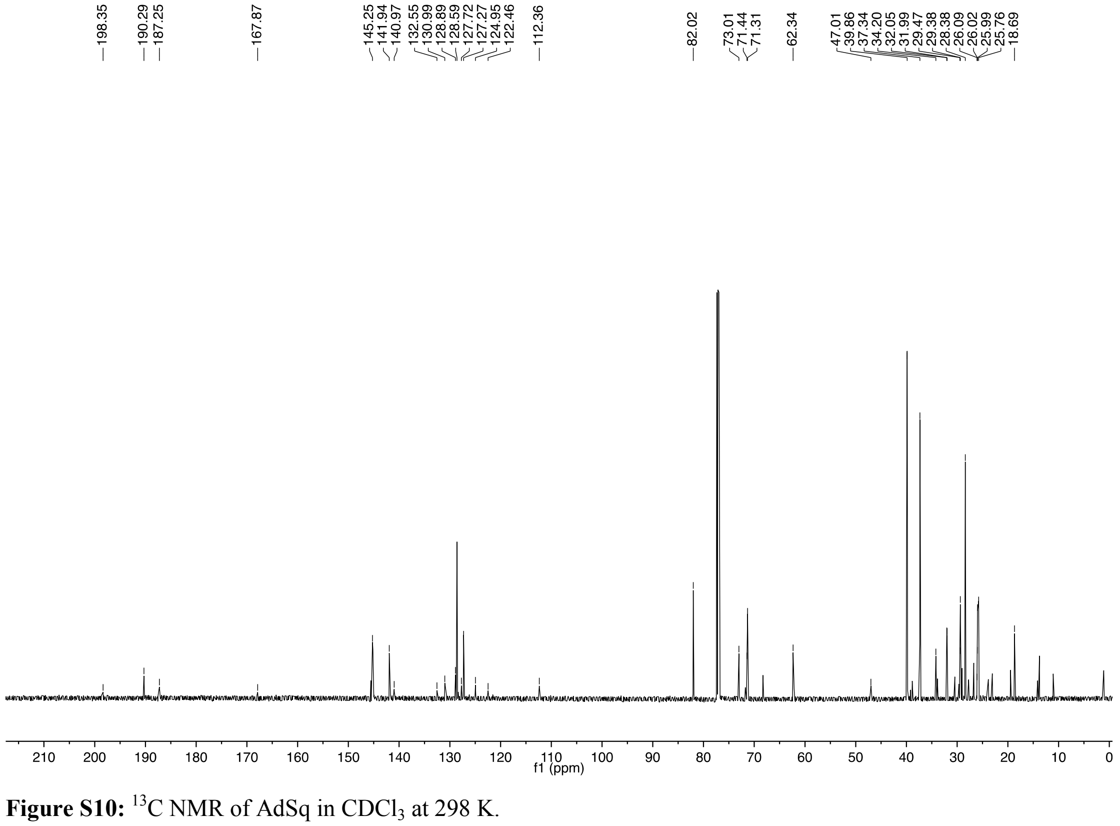

Figure S10: ^{13}C NMR of AdSq in $CDCl_3$ at 298 K.

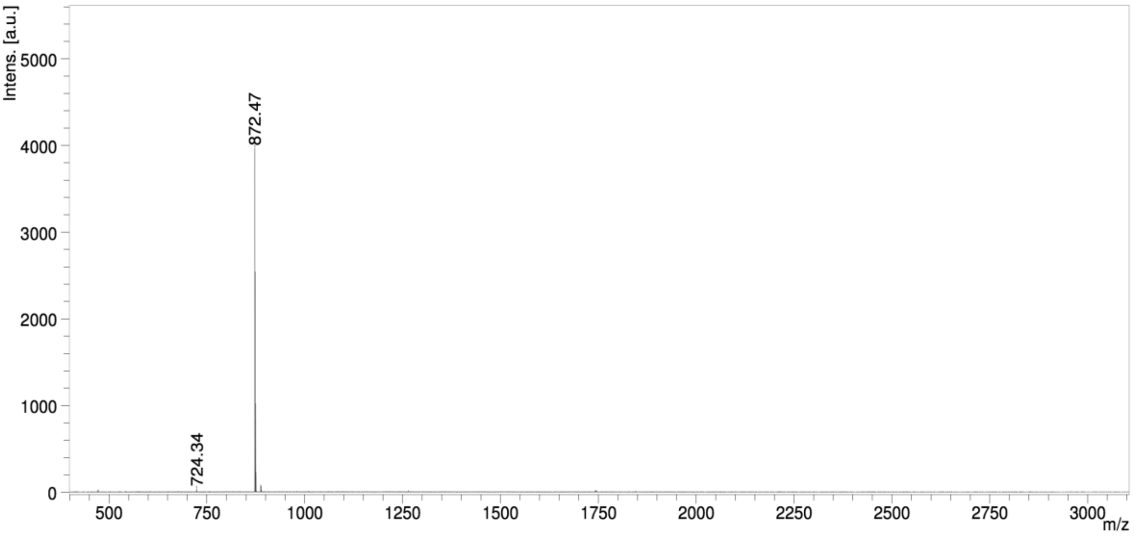

Figure 11: MALDI-mass spectrum of AdSq.
